# High-resolution chromosome painting with repetitive and single-copy oligonucleotides in *Arachis species* identifies structural rearrangements and genome differentiation

**DOI:** 10.1186/s12870-018-1468-1

**Published:** 2018-10-17

**Authors:** Pei Du, Lina Li, Hua Liu, Liuyang Fu, Li Qin, Zhongxin Zhang, Caihong Cui, Ziqi Sun, Suoyi Han, Jing Xu, Xiaodong Dai, Bingyan Huang, Wenzhao Dong, Fengshou Tang, Lifang Zhuang, Yonghua Han, Zengjun Qi, Xinyou Zhang

**Affiliations:** 1 Industrial Crops Research Institute, Henan Academy of Agricultural Sciences/Key Laboratory of Oil Crops in Huang-Huai-Hai Plains, Ministry of Agriculture/Henan Provincial Key Laboratory for Oil Crops Improvement, Zhengzhou, 450002 Henan China; 2 State Key Laboratory of Crop Genetics and Germplasm Enhancement, Nanjing Agricultural University, Nanjing, 210095 China; 3 Jiangsu Key Laboratory of Phylogenomics and Comparative Genomics, School of Life Sciences, Jiangsu Normal University, Xuzhou, 221116 China; 4 School of Life Sciences, Zhengzhou University, Zhengzhou, 450001 Henan China

**Keywords:** *Arachis* species, Chromosome painting, Genomic evolution, High-resolution karyotype, Oligonucleotide multiplex

## Abstract

**Background:**

*Arachis* contains 80 species that carry many beneficial genes that can be utilized in the genetic improvement of peanut (*Arachis hypogaea* L. 2n = 4x = 40, genome AABB). Chromosome engineering is a powerful technique by which these genes can be transferred and utilized in cultivated peanut. However, their small chromosomes and insufficient cytological markers have made chromosome identification and studies relating to genome evolution quite difficult. The development of efficient cytological markers or probes is very necessary for both chromosome engineering and genome discrimination in cultivated peanut.

**Results:**

A simple and efficient oligonucleotide multiplex probe to distinguish genomes, chromosomes, and chromosomal aberrations of peanut was developed based on eight single-stranded oligonucleotides (SSONs) derived from repetitive sequences. High-resolution karyotypes of 16 *Arachis* species, two interspecific F_1_ hybrids, and one radiation-induced M_1_ plant were then developed by fluorescence in situ hybridization (FISH) using oligonucleotide multiplex, 45S and 5S rDNAs, and genomic in situ hybridization (GISH) using total genomic DNA of *A. duranensis* (2*n* = 2*x* = 20, AA) and *A. ipaënsis* (2*n* = 2*x* = 20, BB) as probes. Genomes, chromosomes, and aberrations were clearly identifiable in the established karyotypes. All eight cultivars had similar karyotypes, whereas the eight wild species exhibited various chromosomal variations. In addition, a chromosome-specific SSON library was developed based on the single-copy sequence of chromosome 6A of *A. duranensis*. In combination with repetitive SSONs and rDNA FISH, the single-copy SSON library was applied to identify the corresponding A3 chromosome in the *A. duranensis* karyotype.

**Conclusions:**

The development of repetitive and single-copy SSON probes for FISH and GISH provides useful tools for the differentiation of chromosomes and identification of structural chromosomal rearrangement. It facilitates the development of high-resolution karyotypes and detection of chromosomal variations in *Arachis* species. To our knowledge, the methodology presented in this study demonstrates for the first time the correlation between a sequenced chromosome region and a cytologically identified chromosome in peanut.

**Electronic supplementary material:**

The online version of this article (10.1186/s12870-018-1468-1) contains supplementary material, which is available to authorized users.

## Background

Cultivated peanut (*Arachis hypogaea* L.) is an allotetraploid species that was evolved from a cross of two wild diploid progenitors, *A. duranensis* and *A. ipaënsis* [1]. It is widely cultivated worldwide as both an oil and cash crop. In 2016, total peanut production around the world was 43,982,063 tons and average yield was 1710.49 kg ha^− 1^ [2]. However, peanut productivity is severely constrained by diseases and pests. The development of resistant cultivars is time-consuming and has been challenged by the existence of quantitative resistance traits and limited resistance resources in the cultivated gene pool [1].

The genus *Arachis* comprises 80 species [1], many of which potentially harbor beneficial genes for peanut improvement. Chromosome engineering is a useful approach by which genes from related species can be transferred and exploited. However, it is difficult to cytologically distinguish chromosomes of wild species because of their small size and the paucity of cytological markers. Therefore, many species, such as *Arachis batizocoi*, *A. cruziana*, *A. krapovickasii*, *A. benensis*, *A. trinitensis*, *A. decora*, *A. palustris,* and *A. praecox* have had incorrectly classified genomes [3, 4]. Staining with 4′, 6-diamidino-2-phenylindole (DAPI) and the use of rDNA probes are major tools that are traditionally used to differentiate peanut chromosomes [5–8]. The application of centromeric and telomeric repeat probes has significantly improved the ability to resolve *Arachis* karyotypes [9, 10]. Zhang et al. [11] identified seven *A. duranensis* bacterial artificial chromosome (BAC) clones that facilitated the differentiation of almost all chromosomes of peanut. Unfortunately, the existing markers were primarily distributed in the centromeric or telomeric regions with limited chromosomal polymorphism. In addition, the preparation of these markers is time-consuming and expensive.

SSONs, short DNA or RNA fragments, can be modified and directly hybridized with chromosomal DNA, thereby potentially replace traditional plasmid clones [12]. Oligopainting, using repetitive and single-copy sequences as probes, is a simple and efficient method that is being increasingly used in a wide variety of organisms, including humans [13], propionibacteria [14], cucumber [15], wheat [16], and maize [17]. Two types of SSON probes are currently available: repetitive and single-copy. Repetitive SSONs can be derived from simple sequence repeats (SSRs), genomic sequence repeats (GSRs), and multi-copy genetic sequences (MCGSs) [18–21]. Single-copy SSONs include single-gene and single-copy genomic oligonucleotides [15, 22]. Repetitive SSONs, generally clustered together on one or several genomes or chromosomes, are frequently used to develop high-resolution karyotypes and identify chromosomes [16, 17, 23]. Single-copy SSONs are usually chromosome-specific, and are thus used to track specific chromosomes or chromosomal fragments and identify homoeologous chromosomes in closely related species [13, 15, 24]. In addition, SSONs can be used for species identification [25], haplotype analysis of homologous chromosomes [26], and RNA analysis of single cells [27, 28].

The availability of genome sequences of peanut and its donor species *A. duranensis* (A-genome) and *A. ipaënsis* (B-genome) [29], has facilitated the development of peanut SSON probes and provided a novel method by which peanut chromosomal structure and genomic evolution could be deciphered. The aim of the present study was to investigate chromosomal structural rearrangement and genome differentiation through high-resolution chromosome painting in the genus *Arachis*. The specific objectives were to: 1) develop repetitive SSON probes using *A. duranensis* genomic sequences, SSRs, and telomeric repeats; 2) utilize multiplex probes for simple and efficient chromosome identification; 3) compare high-resolution karyotypes of eight commercial cultivars, eight wild species, two interspecific F_1_ hybrids, and one radiation-induced M_1_ plant to reveal chromosomal rearrangements and genomic evolution; and 4) construct a chromosome-specific single-copy SSON library from the sequenced chromosome 6A to identify the corresponding chromosome in the karyotype.

## Results

### Development of repetitive SSON probes

**Fig. 1:**
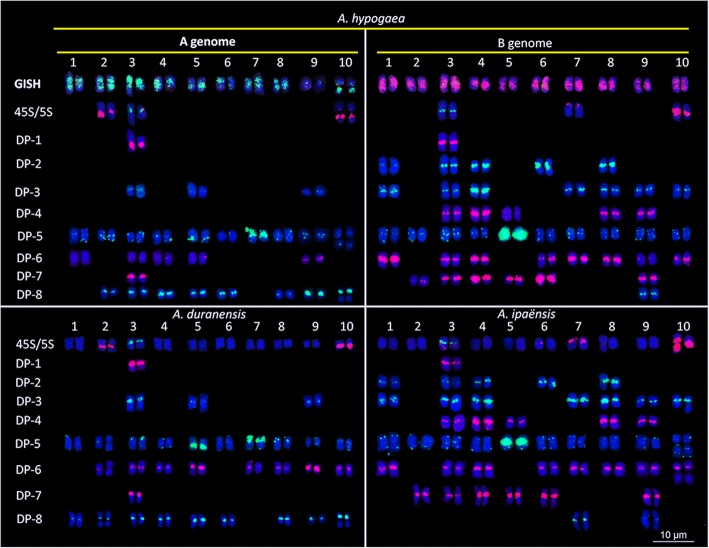
FISH mapping of DP-1 (red), DP-2 (green), DP-3 (green), DP-4 (red), DP-5 (green), DP-6 (red), DP-7 (red), and DP-8 (green) by sequential FISH using 45S rDNA (red), 5S rDNA (green), and *A. duranensis* (green) and *A. ipaënsis* (red) total genomic DNA as probes in *Arachis hypogea* cv. SLH, *A. duranensis*, and *A. ipaënsis.* Blue color represents chromosomes counterstained with DAPI

Eight repetitive SSON probes were identified and designed from different repetitive sequence sources. Probe sequences and the source of repetitive sequences are listed in Table 1. Sequential FISH/GISH was performed to position these SSONs on chromosomes of Silihong (SLH), *A. duranensis* (the A-genome donor), and *A. ipaënsis* (the B-genome donor). All repetitive SSON probes generated distinguishable, stable, and clear signals on chromosomes of SLH, *A. duranensis*, and *A. ipaënsis* (Fig. 1 and Additional file 1: Figure S1 Additional file 2: Figure S2, Additional file 3: Figure S3, Additional file 4: Figure S4). The DP-1 probe produced signals that completely overlapped with those produced by 5S rDNA plasmid clones in SLH (Fig. 1 and Additional file 1: Figure S1). Signals from DP-5 were distributed at the chromosome ends and in the centromeric, pericentromeric, and intercalary regions. They particularly formed larger clusters of strong signals in the long arm of A^hy^5 and the short arm of A^hy^7 of the A-genome, and in the centromeric region of B^hy^5 of the B-genome (Fig. 1; Additional file 3: Figure S3). The DP-6 probe produced signals in 13 chromosomes of SLH. With the exception of one signal, which was detected on the long arm of chromosome A^hy^1, all other signals were found in the centromeric regions of A- and B-genome chromosomes with various intensities. Most of the clear signals of DP-2, DP-3, DP-4, and DP-7 were observed only in the centromeric regions of B-genome chromosomes (Fig. 1). However, one exception was observed for DP-7, which revealed a clear signal in chromosome A3 near the 5S rDNA region, whereas an absence of, or sporadically weak signals were detected on the other A-genome chromosomes. In contrast to the four former SSONs, DP-8 only produced signals on eight A-genome chromosomes and on one B-genome chromosome in SLH (Fig. 1 and Additional file 4: Figure S4).

**Table 1:** Single-stranded oligonucleotide (SSON) probes developed in this study

SSON	Sequence and 5′- end modification	Origin
DP-1	TAMRA-5′-TGCGATCATACCAGCACTAATGCACCGGATCCCGTCAGAACTCTGAAGTTAAGCGTG − 3′	5S rDNA (GenBank: M10470.1)
DP-2	FAM-5′-ACTACTACTACTACTACTACTACTACTACT-3′	(ACT)10
DP-3	FAM-5′- CATTAAATCAGTTATAGTTTGTTTGATGGTA-3′	Genomic DNA
DP-3	TAMRA −5′- CATTAAATCAGTTATAGTTTGTTTGATGGTA-3′	Genomic DNA
DP-4	TAMRA-5′-ATTATTATTATTATTATTATTATTATTATT-3′	(ATT)10
DP-5	FAM-5′-TTTAGGGTTTAGGGTTTAGGGTTTAGGGTTTAGGGTTTAGGGTTTAGGGTTTAGGG-3′	Telomere repeats
DP-6	TAMRA-5′-AAAAAATCGGAGGAGCCTGCCGAAGATGAGG-3′	Genomic DNA
DP-7	FAM −5′-AAAAAAAAAAAAAAAAAAAAAAAAAAAAAAA-3′	Genomic DNA
DP-7	TAMRA-5′-AAAAAAAAAAAAAAAAAAAAAAAAAAAAAAA-3′	Genomic DNA
DP-8	FAM −5′-TGAAAACTTTTTATTTTTAAATTTTGAAACT-3′	Genomic DNA
DP-8	TAMRA-5′-TGAAAACTTTTTATTTTTAAATTTTGAAACT-3′	Genomic DNA

The eight SSON probes produced almost identical signals between the B^i^- and B^hy^-genome chromosomes of the wild donor *A. ipaënsis* and SLH. The only difference was that DP-8 produced a signal only on chromosome B^hy^9 of SLH, but produced an additional signal on B^i^7 in *A. ipaënsis*. Most signals generated by these eight probes were also similar between the A-genome chromosomes of *A. duranensis* and SLH, with only few exceptions. For instance, the signal of DP-5 at the interstitial position on the long arm of A^hy^5 was weaker than that on A^du^5. The DP-6 probe produced signals at centromeric positions on the chromosomes of both species (A3, A4, A5, and A9), and other signals in SLH only (A^hy^1), or *A. duranensis* only (A^du^2, Adu7, A^du^8, and A^du^10), or produced no signal at all (A^du^1).

### High-resolution karyotyping of peanut based on oligopainting using a repetitive SSON multiplex probe

**Fig. 2:**
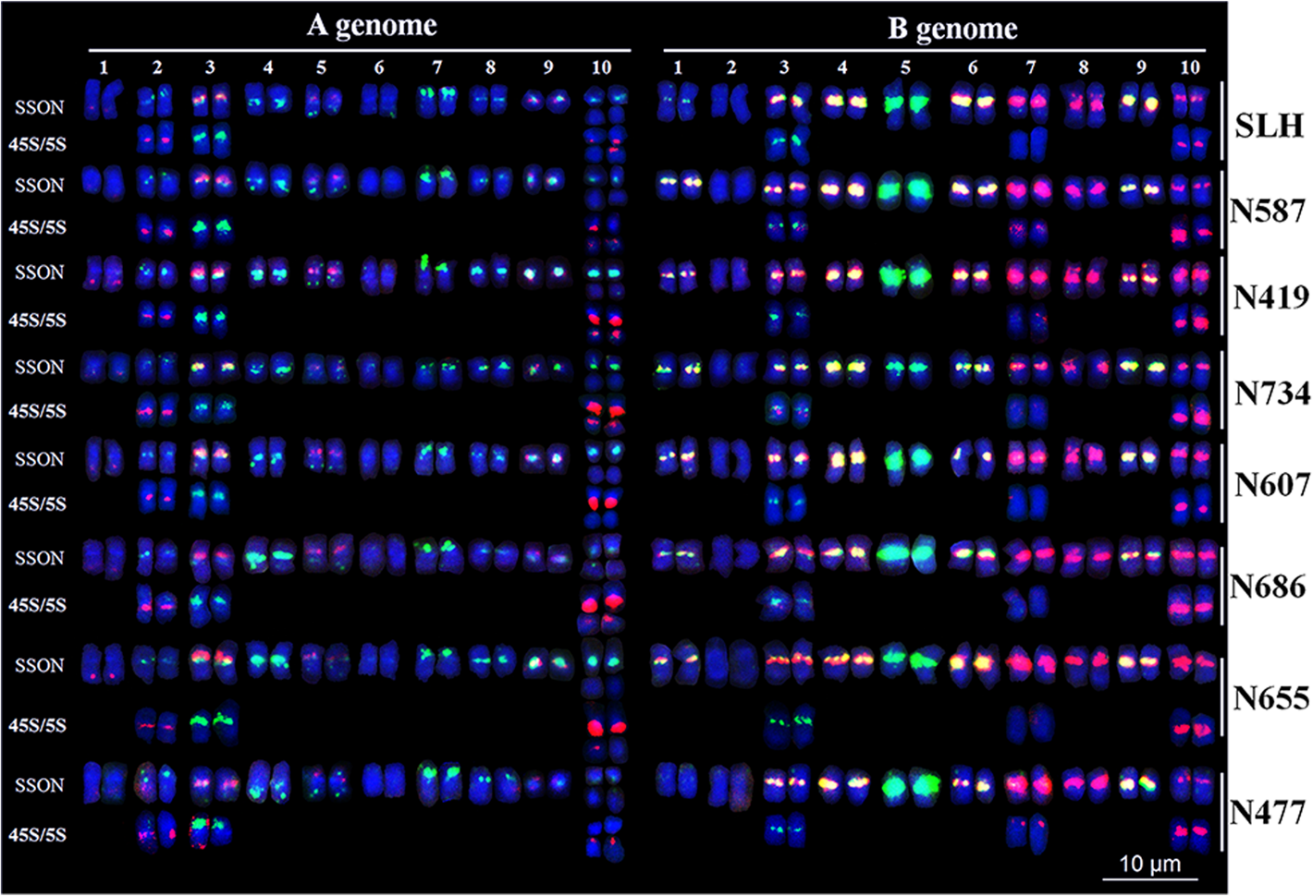
Karyotypes of eight peanut varieties. Lines labeled as SSON and 45S/5S refer to signals from Multiplex #1 and rDNAs, respectively. Blue color represents chromosomes counterstained with DAPI. In SSON, red represents signals of DP-1, DP-3, DP-4, and DP-6; and green represents signals of DP-2, DP-5, and DP-7. In 45S/5S, red represents signals of 45S rDNA and green represents signals of 5S rDNA

A multiplex #1 repetitive SSON probe was developed according to the signal patterns of seven repetitive SSON probes that included 6-carboxyfluorescein (FAM) modified DP-2, 5, and 7, and 6-carboxytetramethylrhodamine (TAMRA) modified DP-1, 3, 4, and 6 (Table 2). Sequential FISH/GISH using Multiplex #1, total genomic DNA of *A. duranensis* and *A. ipaënsis*, and 45S and 5S rDNA probes facilitated the development of a high-resolution karyotype for SLH (Additional file 5: Figure S5). Significant differences in A^hy^ and B^hy^ chromosomes were visible in this karyotype. Among B^hy^ chromosomes, B^hy^5 displayed distinct green centromeric signals, whereas B^hy^8 and B^hy^10 exhibited strong red centromeric signals. Both red and green signals were detected in the centromeric regions of all remaining chromosomes, with the exception of B^hy^2, which displayed no signals. Strong signals of both colors were detected on B^hy^3, B^hy^4, B^hy^6, and B^hy^9, whereas weaker signals of both colors were detected on B^hy^1, and only strong red signal was detected on B^hy^7. Signals of 45S or 5S rDNA were detected on B^hy^3, B^hy^7, and B^hy^10. Among the A^hy^ chromosomes, A^hy^7 and A^hy^5, quite unlike the other A^hy^ chromosomes, showed strong interstitial telomeric signals, on the short arm of A^hy^7 and on the long arm of A^hy^5. Signals associated with both 45S and 5S rDNA loci were detected on A^hy^2, A^hy^3, and A^hy^10. Furthermore, A^hy^6 displayed no signals, A^hy^4 and A^hy^8 displayed large green centromeric signals, and small red signals were detected on the long arm of A^hy^1 (Fig. 2, Additional file 5: Figure S5 and Additional file 6: Figure S6).

**Table 2:** Single-stranded oligonucleotide (SSON) multiplexes developed in this study

Multiplex	SSON with 5′- end modified	Concentration (ng/μL)
#1	FAM - DP-2	100
	FAM - DP-5	100
	FAM - DP-7	26.67
	TAMRA - DP-1	33.33
	TAMRA - DP-3	100.00
	TAMRA - DP-4	100.00
	TAMRA - DP-6	100.00
#2	FAM - DP-2	100
	FAM - DP-5	100
	FAM - DP-7	26.67
	TAMRA - DP-8	26.67

To validate the potential of Multiplex #1, seven other cultivars (N686, N607, N655, N734, N477, N587, and N419) were analyzed. In general, almost all chromosomes of these cultivars were similar to those of SLH, and the karyotypes of all cultivars remained conservative, with the exception of chromosomes A^hy^10 and B^hy^1, both of which showed minor variations in the centromeric and 45S rDNA regions of different cultivars (Fig. 2).

### Chromosomal structural rearrangements revealed in wild *Arachis* species

**Fig. 3:**
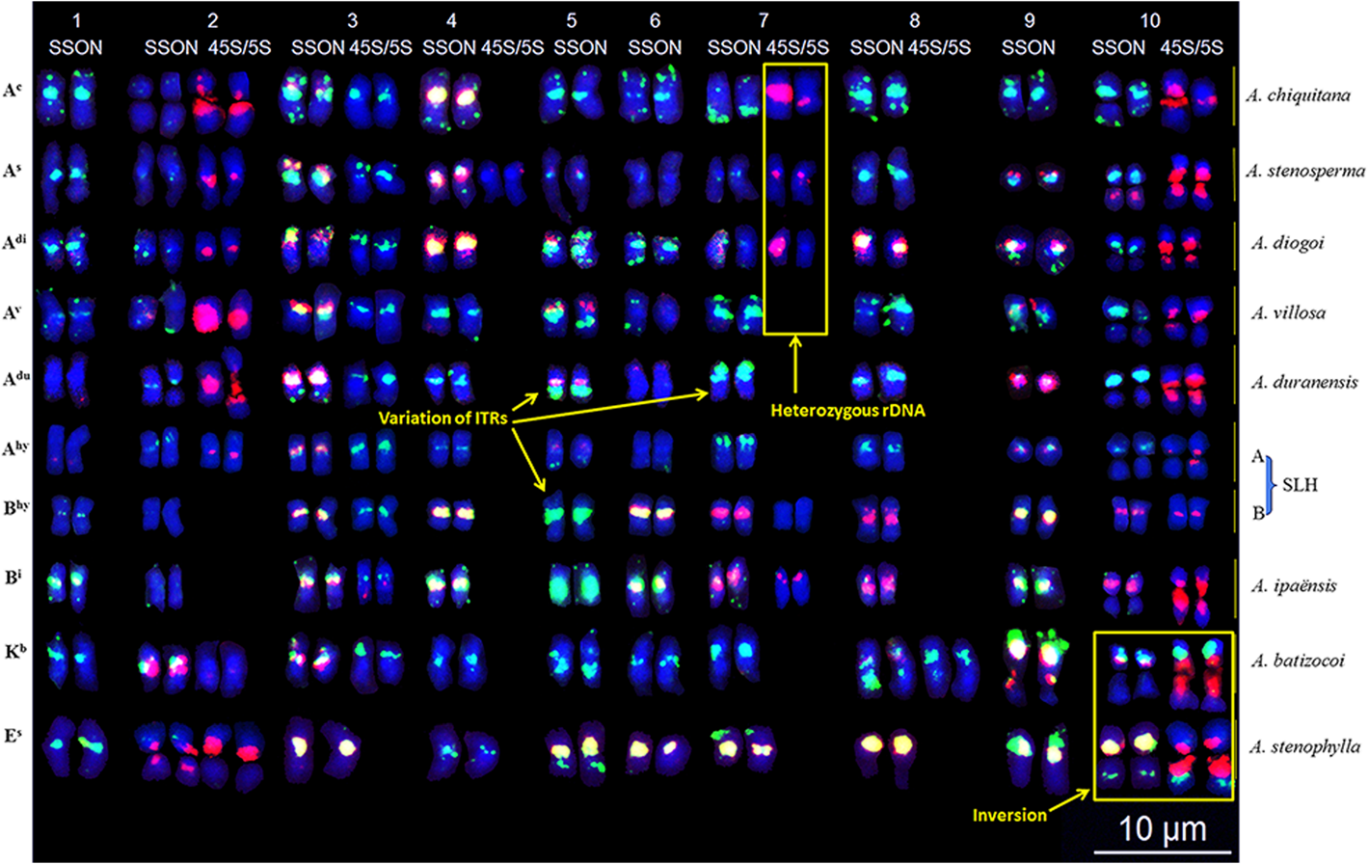
Karyotypes of nine *Arachis* species. Columns labeled as SSON and 45S/5S refer to signals from Multiplex #1 and rDNAs, respectively. Probe color same as Fig. 2

To assay genomic differentiation and chromosomal rearrangements, the chromosomes of eight wild species were discriminated by sequential FISH/GISH using Multiplex #1 (Additional file 6: Figure S6 and Additional file 7: Figure S7). High-resolution karyotypes (Fig. 3) and idiograms of these species (Additional file 8: Figure S8) were developed and compared. Among these species, the genome of *A. duranensis* and *A. ipaënsis* showed a high similarity to the respective A and B genomes of SLH; however, a greater number of green signals were observed on the long arm of A^du^5 than on A^hy^5 (Fig. 3 and Additional file 8: Figure S8).

Significant differences were observed on chromosomes 1, 4, 5, 6, 7, 8, 9, and 10 of six A-genome species (SLH, *A. duranensis*, *A. chiquitana*, *A. stenosperma*, *A. diogoi,* and *A. villosa*). Signals were absent from chromosomes A^hy^1, A^du^1, A^s^6, A^v^6, and A^du^6, whereas green signals were present on the other chromosomes. Chromosomes A^hy^4, A^du^4, A^v^4, A^hy^8, A^du^8, A^v^8, A^s^8, and A^c^8 exhibited green signals only, whereas A^c^4, A^s^4, A^di^4, and A^di^8 displayed both red and green signals. With the exception of the much larger chromosome, A^c^9, which exhibited only green signals, chromosomes A9 among the other species were similar in size and displayed both green and red signals (Fig. 3 and Additional file 8: Figure S8). Green signals were absent from the long arm of chromosomes A^s^5 and A^c^5, and red signals were absent from the centromere of A^c^5. On chromosomes A7, green signals were detected at the ends of the short arms of A^hy^7 and A^du^7, the end of the long arm of A^c^7, and in the centromeric region of A^v^7. Red signals were observed on chromosome A^s^10 at the centromere. Based on these signal patterns, the genome of *A. duranensis* was evidently most similar to the A^hy^ chromosomes of SLH, followed by *A. villosa*, *A. stenosperm*, *A. diogoi*, and *A. chiquitana* (Fig. 3 and Additional file 8: Figure S8).

Karyotypes of *A. batizocoi* and *A. stenophylla* accessions were significantly different from the A and B genomes. In both species, green signals covered almost the entire short arm of chromosome 9, and 45S and 5S rDNA loci were detected on chromosome 10. The chromosomes of *A. batizocoi* and *A. stenophylla* were quite different from each other; *A. batizocoi* displayed 5S rDNA signals on K^b^3 and K^b^8, whereas *A. stenophylla* did not; E^S^5, E^S^6, and E^S^7 exhibited both red and green signals in their centromeric regions, whereas only green signals were observed in these regions on K^b^5, K^b^6, and K^b^7. Furthermore, the distribution of 45S and 5S rDNA loci showed opposite trends between K^b^10 and E^S^10, suggesting an inversion in one of the two species (Fig. 3 and Additional file 8: Figure S8).

Comparative analysis of all karyotypes revealed three types of structural rearrangements in the wild species studied. First, heterogeneous rDNA loci were found in different homologous chromosome pairs. For example, 45S rDNA signals differed between the homologous chromosomes 7 in species of the A-genome. In particular, the intensity of the 45S rDNA signals were identical in A^s^7 and differed in A^c^7. The 45S rDNA signal was detected only on the A^di^7 chromosome; however, these signals were lacking on A^du^7 (Fig. 3 and Additional file 8: Figure S8). Second, interstitial telomeric signal positions and amplifications were variable. Signals of different intensities and sizes were found at the chromosome ends (A^du^7 and E^s^9), in the middle of chromosome arms (A^du^5), or near centromeres (B^hy^5) (Fig. 3 and Additional file 8: Figure S8). Third, chromosome morphology was variable among different species. For example, chromosomes A^c^9 and A^s^9 were small and metacentric; whereas E^s^9 was submetacentric and E^s^8 was subtelocentric (Additional file 8: Figure S8).

### Chromosomal characterization of interspecific F_1_ hybrids and an irradiation-induced M_1_ plant

**Fig. 4:**
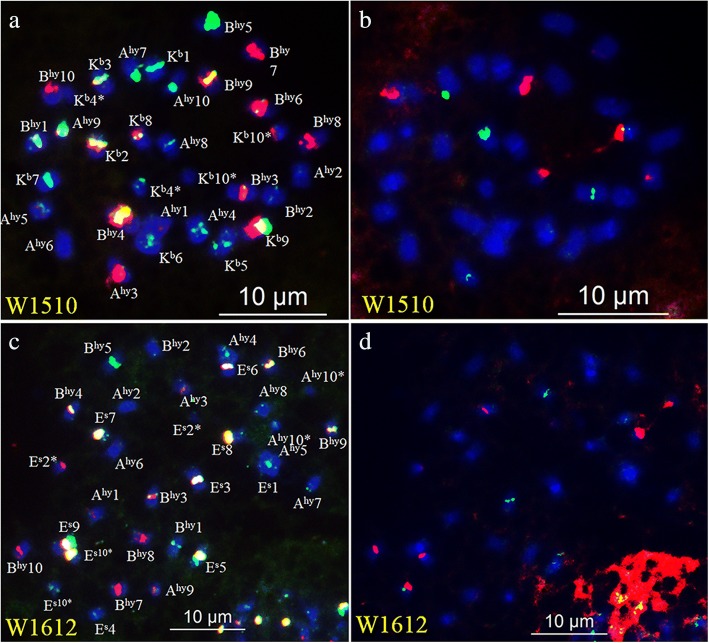
Sequential FISH of two hybrids F_1_ w1510 and w1612 using Multiplex #1 (**a** and **c**), and 5S and 45S rDNA (**b** and **d**) plasmid clones as probes. **a**–**b**: w1510 derived from SLH (*A. hypogaea*) and *A. batizocoi*; **c**–**d**: w1612 derived from N734 (*A. hypogaea*) and *A. stenophylla*. Probe color as Fig. 2

To further validate the potential of Multiplex #1, two interspecific F_1_ hybrids were examined by sequential FISH/GISH. Two genome sets of the hybrid w1510 were identical to A^hy^ and B^hy^ of SLH, and the third genome set was the same as K^b^ of *A. batizocoi* (Fig. 4 and Additional file 9: Figure S9). For w1612, two genome sets were identical to A^hy^ and B^hy^ of N734, and the third was similar to E^S^ of *A. stenophylla* (Fig. 4 and Additional file 9: Figure S9). These results indicate that both w1510 and w1612 were true F_1_ hybrids.

**Fig. 5:**
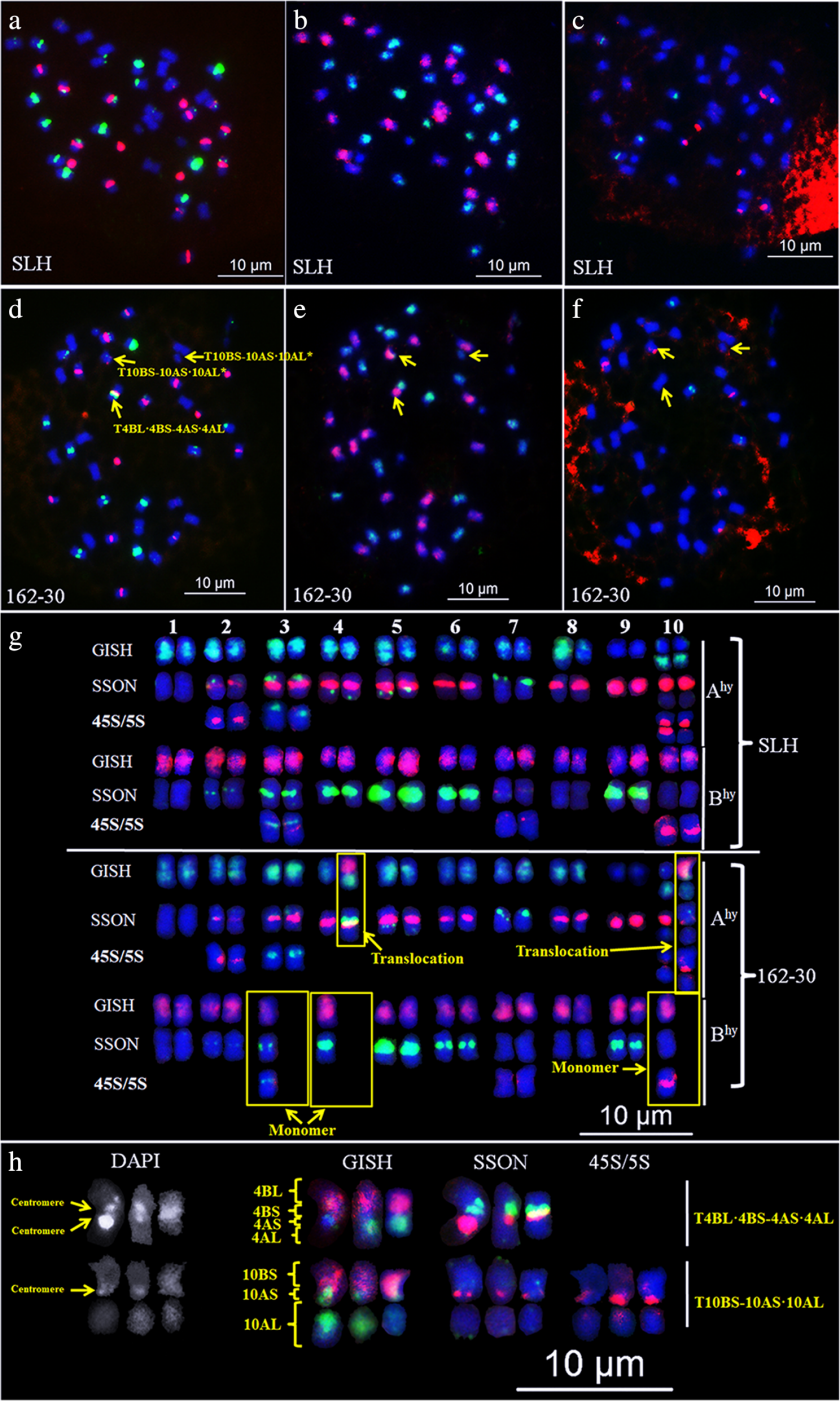
Chromosome aberrations detected in radiation-induced M_1_ plant 162–30 of peanut cultivar SLH after sequential FISH/GISH. **a**–**f** Results of sequential FISH/GISH in SLH (**a**–**c**) and M1 plant 162–30 (**d**–**f**) using Multiplex #2 (**a** and **d**), *A. duranensis* genomic DNA (**b** and **e**; green), *A. ipaënsis* genomic DNA (**b** and **e**; red), 5S rDNA (**c** and **f**; green), and 45S rDNA (**c** and **f**; red). **g** Karyotypes of SLH and 162–30. **h** Translocated chromosomes in 162–30. Multiplex #2 contains four SSONs, including TAMRA-DP-8, FAM-DP-2, FAM-DP-5, and FAM-DP-7

Multiplex #2, which included TAMRA-modified DP-8, and FAM-modified DP-2, DP-5, and DP-7, was used for oligopainting with sequential GISH/FISH to characterize a radiation-induced M_1_ plant (162–30). By applying this probe, we were able to identify two chromosomal translocations and one monosomic chromosome (Fig. 5a ~ 5f). One translocation between A^hy^4 and B^hy^4 that contained two centromeric DAPI bands and SSON centromeric bands of both colors, formed a potential dicentric chromosome T4BL·4BS-4AS·4AL. The other translocation, T10BS-10AS·10AL, involved a large satellite. The monosomic chromosome was identified in the B^hy^3 chromosome because of its unique signal patterns (Fig. 5g and h).

### Oligopainting using the single-copy sequence library of chromosome 6A as probes, decorated the cytologically designated chromosome A3 of *A. duranensis*

**Fig. 6:**
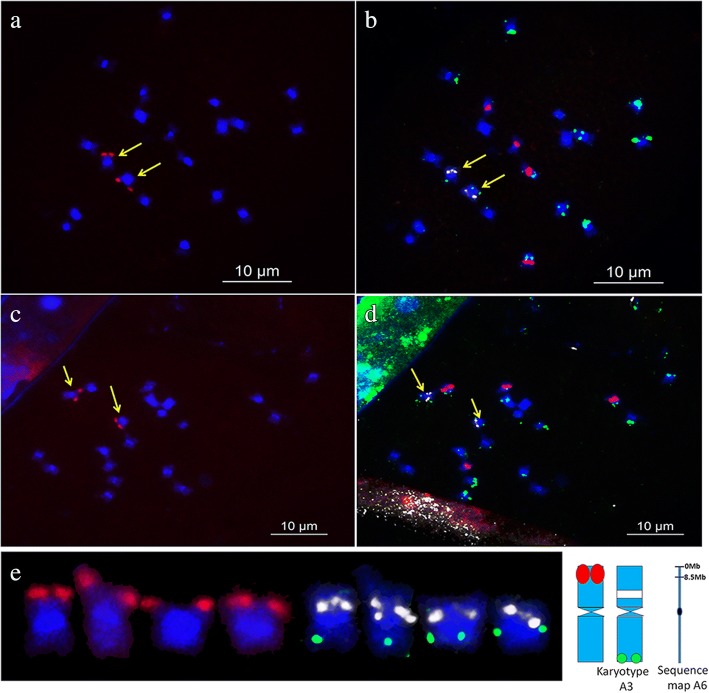
Oligopainting using single-copy oligonucleotide library 6A-1 combined with sequential FISH using DP-5, and plasmid clones 45S rDNA and 5S rDNA as probes in *A. duranensis*. Blue color represents chromosomes counterstained with DAPI; **a** and **c** shows oligopainting using single-copy SSON library 6A-1 (green); **b** and **d**, sequential FISH using repetitive SSON DP-5 (green), 45S rDNA (red), and 5S rDNA (white inverted from the original green); **c** From left to right: chromosome with signals of library 6A-1 (green); karyotype of A3, probe signals are same as (**b**) and (**d**); idiograms of the chromosome displaying signals from library 6A-1 and the karyotype of A3; and sequence map of A6 showing 0–8.5 Mb regions corresponding to library 6A-1. Yellow arrows in panels (**a**–**d**) indicate chromosomes with signals from library 6A-1

To correlate a sequenced chromosome with a cytologically identified chromosome, a single-copy SSON library was developed from a ~ 8.5 Mb distal region of 6A and used to paint metaphase chromosomes of *A. duranensis*. A pair of clear signals was reputably detected on one pair of chromosomes of *A. duranensis* (Additional file 10: Figure S10). Sequential FISH using 45S, 5S rDNA, and DP-5 probes (Additional file 11: Figure S11) revealed that the target signals were present in cytologically designated chromosome A3, which displayed a typical distribution of 5S rDNA on its short arm (Fig. 6).

## Discussion

### Oligopainting using repetitive and single-copy sequences as probes provides a simple and efficient method for chromosome identification in peanut

Chromosome identification has previously been carried out in peanut using primarily FISH with 45S and 5S rDNA probes and DAPI staining [6–8]. Zhang et al. [11] recently developed seven BAC clones in *Arachis* as powerful new markers for chromosome identification. In the present study, eight repetitive SSON probes and one single-copy SSON library were successfully developed, and the repetitive SSON probes were combined into two powerful multiplexes (Multiplex #1 and Multiplex #2) for efficient chromosome discrimination. The two multiplexes effectively differentiated the chromosomes and genomes of eight peanut cultivars, eight wild species, two interspecific hybrids, and one radiation-induced M_1_ plant, and revealed structural rearrangements in these species and accessions. Repetitive SSON probes for the identification of peanut chromosomes can be easily designed and modified, thus greatly reducing the required time and expense. The repetitive SSON probes can be used advantageously in nondenaturing FISH, thereby further simplifying the FISH procedure [16]. Our findings demonstrated that repetitive SSON probes could be useful and provide powerful tools for peanut chromosome engineering. However, the current SSONs did not cover all chromosome regions and involved only limited types of repetitive sequences. A further study should utilize various sources of repetitive sequences to identify specific chromosomes or regions.

The International Peanut Sequencing Project has sequenced the reference genomes of *A. duranensis* and *A. ipaënsis* [29]. However, no direct correspondence has been established between the peanut linkage map and cytologically identified chromosomes. Based on the genome sequence of chromosome A6 of *A. duranensis*, we successfully developed a chromosome-specific single-copy SSON library, which could be used to paint the target chromosomes. By using both the repetitive SSONs and single-copy sequences as probes for oligopainting, we identified the target chromosomes and successfully correlated a sequenced A6 segment to the actual chromosome. This opens a path to further correlation of all sequenced chromosomes with cytologically identified chromosomes. The development of more single-copy SSON libraries for each chromosome of peanut is underway at our institute.

### New clues for genomic evolution revealed by painting of *Arachis species*

In the cultivated peanut and its wild ancestors, chromosome painting with the eight probes generated fewer and weaker signals on A-genome chromosomes than on those of the B-genome. For example, three SSON probes, DP-2, − 4, and − 7, derived from SSRs (ACT)_10_, (ATT)_10_, and (A)_31_, respectively, rarely produced signals on A-genome chromosomes, but yielded strong signals on chromosomes of the B-genome, which showed more SSRs variations in *A. ipaënsis* than in *A. duranensis*. These results are consistent with the fact that although mono-, di-, and tri-nucleotides are the most abundant motifs in *A. duranensis* and *A. ipaënsis*, a greater number of SSRs are present in *A. ipaënsis* than in *A. duranensis* [30]. In addition, *A. ipaënsis* contains more transposable elements than *A. duranensis* does [29]. This suggests that the B-genome has more repeats, and may explain why the size of B-genome is larger than that of the A-genome (1.56 Gb vs 1.25 Gb) [29]. Other repetitive SSONs also produced weaker and fewer signals on A chromosomes, in comparison to B chromosomes. For example, signals between B^i^ and B^hy^ genomes, with the exception of DP-8 on chromosome 7, were almost identical in terms of position and intensity; however, fewer signals were present on four chromosomes of the A^hy^ genome relative to A^du^. Moreover, some signals on the A^hy^ genome, such as A^hy^5, varied according to their quantity and position (Fig. 1), suggesting that during evolution, large chromosomal rearrangements occurred in the A-genome chromosomes rather than in the B-genome chromosomes. These results correspond well with the findings of Bertioli et al. [29] and Zhang et al. [11].

Although the eight tested cultivars represented three different peanut taxa (varieties*. Fastigiata*, *hypogea*, and *vulgaris*), their karyotypes were similar to each other, even though a signal variation was found between chromosomes A^hy^10 and B^hy^1. This observation provides evidence that these cultivated peanut varieties arose from a single allotetraploid species [31].

Our results indicated that the genome of *A. duranensis* was most similar to the A-genome of the cultivated peanut, followed by the genomes of *A. villosa*, *A. stenosperma*, *A. diogoi*, and *A. chiquitana*, supporting the contention that *A. duranensis* was the donor of the A-genome [7]. Similarly, the identical karyotypes observed between *A. ipaënsis* and the B-genome of cultivated peanut supports the notion of *A. ipaënsis* as the B-genome donor [3, 8, 29, 32].

Chromosome identification by oligopainting likewise facilitates the classification of species. *A. chiquitana* was originally grouped with P genome species [33]. However, because it carried a small “A chromosome” and showed similar 45S rDNA, 5S rDNA, and DAPI banding patterns to those of A-genome species, it was regrouped with species of the A-genome [7]. In the present study, the karyotype of *A. chiquitana* was considerably different from those of other species. For example, the morphology and repetitive SSON banding pattern of A^c^9 were very distinct from those of A9 chromosomes of other species with the A-genome. The genome of *A. chiquitana* thus does not seem to be associated with the species group bearing the A-genome. Genomes E^S^ and K^b^ are more closely related to each other than they are to other genomes, even though they belong to different sections of the genus *Arachis*.

Comparative karyotyping by SSON DP-5 revealed that variable internal telomeric repeats (ITRs) exist in chromosomes. They might have descended from telomere-mediated chromosomal rearrangements in these repeats during genomic evolution in peanut. Heterogeneous ITRs have also been observed in tomato, and some ITRs were evidently extremely amplified [34]. Chromosome 10 of *A. stenophylla* and *A. batizocoi* both contained an obvious inversion in the region containing 45S and 5S rDNA loci, providing further evidence for chromosomal structural rearrangements.

From both our previous report [10] using the PCR-amplified telomeric repeats as probes, and the present results using multiple oligonucleotide probes, the discovery of heterogeneous distributions of rDNAs, chromosome inversions, duplications, and morphological variations in different peanut genomes has indicated that *Arachis* might have undergone significant chromosomal rearrangements during genome evolution. Further oligopainting using a greater number of repetitive and single-copy SSON probes should allow us to gain more information on these changes.

## Conclusions

Repetitive and single-copy SSONs and multiplex repetitive SSON probes developed in the present study can be useful tools for simple and efficient chromosome identification and genomic differentiation, and facilitate chromosome engineering in peanut. Oligopainting using repetitive and single-copy sequences as probes showed that modern peanut cultivars have almost identical karyotypes, indicating low genetic diversity. In contrast, wild species exhibited an abundance of chromosomal rearrangements and variations, suggesting their genomic potential for peanut improvement. With the availability of refined reference genome sequences of peanut, additional repetitive and single-copy SSON probes can be developed and used to correlate genetic and cytological maps. Further chromosomal rearrangements, including translocations, inversions, and duplications, should be identifiable using these types of probes. The repetitive and single-copy SSON probes and methodology described in the present study provide useful tools for further application and cytogenetic study in peanut.

## Methods

### Plant materials

Eight accessions of cultivated peanut (*A. hypogaea*) (2*n* = 2*x* = 40) and eight diploid *Arachis* species (*A. chiquitana*, *A. stenosperma*, *A. diogoi*, *A. villosa*, *A. duranensis*, *A. ipaënsis*, *A. batizocoi*, and *A. stenophylla*) were used in this study. Five cultivars (SLH, Kainong30, Linguidachezi, Yuhua15, Liuchengzhenzhudou) were from Henan Academy of Agricultural Sciences. The other three cultivars and eight diploid species were introduced from National Germplasm Resources Laboratory of USA (Beltsville, MD). Accession number, chromosome number, genomic constitution, origin and identification of each plant material are listed in Table 3. In addition, two interspecific F_1_ hybrids (w1510 from a cross between the SLH variety and *A. batizocoi*, and w1612 from a cross between the N734 variety and *A. stenophylla*), and one irradiation-induced SLH M_1_ plant (designated as 162–30) generated by pollen irradiation at a dosage of 16 Gy using a ^60^Co source (Isotope Institute Co. Ltd., Henan Academy of Sciences) were also used as plant materials.

**Table 3:** Plant materials used in this study

Accession number	PI number or variety name	Identification		Origin	Section (genome)	Species (type)	References
		Collector	No.				
SLH	Silihong	Zhang XY		China	*Arachis* (A^hy^A^hy^B^hy^B^hy^)	*A. hypogaea* (*var. fastigiata*)	
N686	PI 158854	Holbrook	GP-134	China	*Arachis* (A^hy^A^hy^B^hy^B^hy^)	*A. hypogaea* (*var. hypogaea*)	[42]
N607	Kainong30	Zhang XY		China	*Arachis* (A^hy^A^hy^B^hy^B^hy^)	*A. hypogaea* (*var. hypogaea*)	
N655	Linguidachezi	Zhang XY		China	*Arachis* (A^hy^A^hy^B^hy^B^hy^)	*A. hypogaea* (*var. hypogaea*)	
N734	Yuhua15	Zhang XY		China	*Arachis* (A^hy^A^hy^B^hy^B^hy^)	*A. hypogaea* (*var. hypogaea*)	
N477	PI 240560	Holbrook	GP-14	South Africa	*Arachis* (A^hy^A^hy^B^hy^B^hy^)	*A. hypogaea*(*var. hypogaea*)	[42]
N587	PI 290560	Holbrook	GP-162	India	*Arachis* (A^hy^A^hy^B^hy^B^hy^)	*A. hypogaea* (*var. fastigiata*)	[42]
N419	Liuchengzhenzhudou	Zhang XY		China	*Arachis* (A^hy^A^hy^B^hy^B^hy^)	*A. hypogaea* (*var. vulgaris*)	
Zw51	PI 338280	Hammons	410	Brazil	*Arachis* (A^s^A^s^)	*A. stenosperma* Krapov. & W. C. Greg.	[1, 7, 43﻿]
Zw52	PI 298639	Krapovickas	9484	Bolivia	*Arachis* (K^b^K^b^)	*A. batizocoi* Krapov. & W. C. Greg.	[3, 43]
Zw53	PI 468322	Krapovickas	30,076	Bolivia	*Arachis* (B^i^B^i^)	*A. ipaensis* Krapov. & W. C. Greg.	[3, 43]
Zw55	PI 219823	Krapovickas	7988	Argentina	*Arachis* (A^du^A^du^)	*A. duranensis* Krapov. & W. C. Greg.	[7, 43]
Zw56	PI 298636	Tweedi	22,585	Argentina	*Arachis* (A^v^A^v^)	*A. villosa* Benth.	[1, 7, 43]
Zw57	PI 276235	Simpson and Charles	10,602	Paraguay	*Arachis* (A^di^A^di^)	*A. diogoi* Hoehne	[1, 7, 43]
Zw59	PI 476006	Krapovickas	36,027	Bolivia	*Arachis* (A^c^A^c^)	*A. chiquitana* Krapov. et al.	[7, 43]
Zw61	PI 468178	Gregory	30,136	Brazil	*Erectoides* (E^s^E^s^)	*A. stenophylla* Krapov. & W. C. Greg.	[1, 43]

### Chromosome preparation

Seeds were germinated for 7 d on moist filter paper at 25 °C. Healthy lateral root tips were excised and pretreated with 2 mM 8-hydroxyquinoline for 3 h at 25 °C, fixed in 3:1 absolute ethanol: glacial acetic acid for 5 h at 25 °C, and stored at − 20 °C. Root meristem sections of 0.3–0.5 mm were excised, squashed in 45% glacial acetic acid, and frozen at − 80 °C for 12 h. The chromosome spreads were dehydrated in 100% ethanol and air-dried after removal of the cover slips.

### Repetitive SSONs and single-copy SSON design and labeling

Eight repetitive SSON probes, named DP-1 to DP-8, were selected from different sources of repetitive sequences. The DP-2 and DP-4 probes were screened from 10 tri-nucleotide SSRs, including (AAC)_10_, (AAG)_10_, (AGG)_10_, (ACT)_10_, (CAT)_10_, (CAC)_10_, (ACG)_10_, (CAG)_10_, (AAT)_10_, and (GCC)_10_, and four di-nucleotide SSRs, namely (AT)_15_, (GC)_15_, (AG)_15_, and (AC)_15_, as described by Cuadrado et al. [18] and Du et al. [16] (Table 1). The design of the DP-5 probe was based on the repeat sequence (TTTAGGG)_n_, as described by Cuadrado et al. [12] and Du et al. [10]. The DP-1 probe was designed by Blasting the wheat 5S rDNA sequence against genomic sequences of *A. duranensis* (Table 1)*.* Whereas DP-3, DP-6, DP-7, and DP-8 were designed following the method of Du et al. [16] using the 4 Gb genomic sequence of *A. duranensis* (unpublished data). The *A. duranensis* genomic sequence *k-mers* were counted using the Jellyfish tool (Version 1.1.11) [35] with the following parameters: *k-mer* = 31 nt and copy number > 1000. The repeat sequences were analyzed by the CD-HIT Suite and DNAMAN software, and those sequences having more than 80% identity with other repeats were discarded. From the remaining 18,052 oligonucleotides, repetitive SSONs with the highest copy number were selected. This selection resulted in 150 repetitive SSONs that were synthesized and labeled by random labeling, and analyzed on SLH chromosomes. Four repetitive SSON probes, DP-3, DP-6, DP-7, and DP-8, were then preferably selected for use in the present study because of their stronger signals. All eight repetitive SSONs were modified at the 5′-ends with TAMRA or FAM by the Invitrogen Company (Shanghai, China) and are listed in Table 1.

Single-copy SSONs were developed according to the method described by Han et al. [15], in which the genomic sequences of chromosome 6A of *A. duranensis* in PeanutBase [36] were downloaded and analyzed. Using the distal ~ 8.5 Mb region of chromosome 6A as a target region, all repetitive sequences were masked with RepeatMasker [37]. The remaining sequences were divided into single-copy SSONs of 42–48 nt in a stepwise size of 5 nt. Single-copy SSONs containing more than 6 nt of homopolymers were discarded. Each single-copy SSON was aligned to the genomic reference sequence of *A. duranensis* using the BLAT tool [38] and screened for homologs with > 75% similarity in the genome. Melting point ™ and hairpin *Tm* values of each single-copy SSON were calculated using the Primer3 software [39]. Single-copy SSONs with d*Tm* > 10 °C (d*Tm* = *Tm* − hairpin *Tm*) were retained for probe construction. As a result, 20,000 selected single-copy SSONs were synthesized by MYcroarray (Ann Arbor, MI, USA). The resulting library was amplified and labeled with digoxigenin-11-dUTP according to the MYcroarry_MYtags labeling protocol [40].

### FISH and sequential FISH analysis

Total genomic DNA was extracted from fresh young leaves of *A. duranensis* and *A. ipaënsis* [41]. Clones of 5S and 45S rDNA of wheat (*Triticum aestivum* L.) were provided by Dr. Bikram S Gill, of Kansas State University, USA. The 5S rDNA clone and total genomic DNA of *A. ipaënsis* were labeled with biotin-16-dUTP (Roche) by nick translation and detected with fluorescein anti-biotin (Roche), whereas 45S rDNA and total genomic DNA of *A. ipaënsis* were labeled with digoxigenin-11-dUTP (Roche) and detected with anti-digoxigenin-rhodamine (Roche).

The FISH procedure followed the method described by Zhu et al. [17]. Briefly, the hybridization solution contained 7.5 μL formamide (Sigma), 1.5 μL 20× saline-sodium citrate (SSC) buffer, 1.0 μL of each probe, 2.0 μL of 50% dextran sulfate (Sigma), and ddH_2_O added to bring the total volume to 15 μL. The hybridization solution was denatured at 105 °C for 13 min, and the spread chromosomes were denatured in 70% formamide at 75 °C for 70 s, and then hybridized overnight at 37 °C. After hybridization, the slides were washed three times in 2× SSC at room temperature, and mounted with VECTASHIELD Mounting Medium, following which DAPI staining was performed.

Sequential FISH/GISH procedures were performed to map the signals of oligonucleotide probes. Briefly, slides were denatured after FISH using repetitive and single-copy SSON probes, and then washed to remove all signals, and subsequently dried. The GISH procedure was performed using total genomic DNA of *A. duranensis* and *A. ipaënsis* as probes to identify A- and B-genome chromosomes, respectively. A second FISH procedure using 45S rDNA and 5S rDNA probes was conducted on the same slides to develop karyotypes, as described by Du et al. [10].

### Fluorescence microscopy and imaging

Slides were examined using a Leica DM6000 fluorescence microscope (Leica). Separate images from each filter set were captured using a cooled CCD camera (Leica). Images were optimized for contrast and brightness using Adobe Photoshop. To distinguish the signals produced in each chromosome by different probes after sequential FISH, the original green signal of 5S rDNA was converted to pseudo-color white. For karyotyping and chromosome diversity analysis, 3–5 cells of each accession, including 6–10 samples of each chromosome, were observed. Most karyotypes were developed from a single cell, unless they were derived from overlapping chromosomes. Chromosomes of each species were primarily ordered based on morphology, size, and unique patterns, as reported by Du et al. [10].

## Additional files

Additional file 1: **Figure S1.** FISH mapping of DP-2 (b, g, and l; green) and DP-1 (c, h, and m; red) by sequential FISH using 45S rDNA (e, g and j; red), 5S rDNA (e, g and j, green), and *A. duranensis* (a, green) and *A. ipaënsis* (a, red) total genomic DNA as probes in SLH (a–e), *A. duranensis* (f–j), and *A. ipaënsis* (k–o). (TIF 2370 kb)

Additional file 2: **Figure S2.** FISH mapping of DP-3 (b, g, and l; green) and DP-4 (c, h, and m; red) by sequential FISH using 45S rDNA (e, g, and j; red), 5S rDNA (e, g, and j, green) and *A. duranensis* (a, green) and *A. ipaënsis* (a, red) total genomic DNA as probes in SLH (a–e), *A. duranensis* (f–j), and *A. ipaënsis* (k–o). (TIF 2188 kb)

Additional file 3: **Figure S3.** FISH mapping of DP-5 (b, g, and l; green) and DP-6 (c, h, and m; red) by sequential FISH using 45S rDNA (e, g, and j; red), 5S rDNA (e, g, and j, green) and *A. duranensis* (a, green) and *A. ipaënsis* (a, red) total genomic DNA as probes in SLH (a–e), *A. duranensis* (f–j), and *A. ipaënsis* (k–o). (TIF 2587 kb)

Additional file 4: **Figure S4.** FISH mapping of DP-8 (b, g, and l; green) and DP-7 (c, h, and m; red) by sequential FISH using 45S rDNA (e, g, and j; red), 5S rDNA (e, g, and j, green) and *A. duranensis* (a, green) and *A. ipaënsis* (a, red) total genomic DNA as probes in SLH (a–e), *A. duranensis* (f–j), and *A. ipaënsis* (k–o). (TIF 2283 kb)

Additional file 5: **Figure S5.** Sequential FISH/GISH using Multiplex #1 (left column, SSON), *A. duranensis* genomic DNA, *A. ipaënsis* genomic DNA (middle column, GISH), 45S rDNA, and 5S rDNA (right column, 45S/5S) as probes in eight peanut varieties. (TIF 2593 kb)

Additional file 6: **Figure S6.** Dual-color FISH using Multiplex #1 in SLH (a ~ d), *A. duranensis* (e ~ h), and *A. ipaënsis* (i ~ l). (TIF 1882 kb)

Additional file 7: **Figure S7.** Sequential FISH using multiplex #1 (left column, SSON), 45S rDNA, and 5S rDNA (right column, 45S/5S) as probes in eight *Arachis* species. (TIF 3606 kb)

Additional file 8: **Figure S8.** Idiogram karyotypes of SLH and eight *Arachis* species. sm, submetacentric; st, subtelocentric; Bar, 3 μm. (TIF 666 kb)

Additional file 9: **Figure S9.** Karyotypes of two hybrid F_1_-derived cultivated peanut and diploid wild species using repetitive Multiplex #1 (SSON), 45S and 5S rDNA (45S/5S) plasmid clones as probes. (TIF 1258 kb)

Additional file 10: **Figure S10.** FISH using oligonucleotide library 6A-1 in *A. duranensis*. (TIF 1066 kb)

Additional file 11: **Figure S11.** Sequential FISH using DP-5 (a and e, green), 45S rDNA (b and f, green), and 5S rDNA (c and g, green) in *A. duranensis*, and its merged figures (d and h). (TIF 1141 kb)

## Abbreviations

BAC: Bacterial artificial chromosome
DAPI: 4′, 6-diamidino-2-phenylindole
FAM: 6-carboxyfluorescein
FISH: Fluorescence in situ hybridization
GISH: Genomic in situ hybridization
GSRs: Genomic sequence repeats
ITRs: Interstitial telomeric repeats. SSC: saline-sodium citrate
MCGSs: Multi-copy genetic sequences
SLH: Silihong
SSONs: Single-stranded oligonucleotides;
SSRs: Simple sequence repeats
TAMRA: 6-carboxytetramethylrhodamine

## References

[1] Stalker HT (2017). Utilizing wild species for peanut improvement. Crop Sci.

[2] Food and Agriculture Organization of the United Nations. http://www.fao.org/faostat/en/#data/QC. Accessed 20 Jan 2018.

[3] Robledo G, Seijo G (2010). Species relationships among the wild B genome of *Arachis* species (section *Arachis*) based on FISH mapping of rDNA loci and heterochromatin detection, a new proposal for genome arrangement. Theor Appl Genet.

[4] Silvestri MC, Ortiz AM, Lavia GI (2015). rDNA loci and heterochromatin positions support a distinct genome type for ‘x = 9 species’ of section *Arachis* (*Arachis*, Leguminosae). Plant Syst Evol.

[5] Raina SN, Mukai Y (1999). Genomic in situ hybridization in *Arachis* (Fabaceae) identifies the diploid wild progenitors of cultivated (*A. hypogaea*) and related wild (*A. monticola*) peanut species. Plant Syst Evol.

[6] Seijo JG, Lavia GI, Fernández A, Krapovickas A, Ducasse D, Mmoscone EA (2004). Physical mapping of the 5S and 18S–25S rRNA genes by FISH as evidence that *Archis duranensis* and *A. ipaënsis* are the wild diploid progenitors of *A. hypogaea* (Leguminosae). Am J Bot.

[7] Robledo G, Lavia GI, Seijo JG (2009). Species relations among wild *Arachis* species with the Agenome as revealed by FISH mapping of rDNA loci and heterochromatin detection. Theor Appl Genet.

[8] Nielen S, Fonseca FC, Bertioli SL, Guimarães P, Seijo G, Town C (2010). FIDEL—a retrovirus-like retrotransposon and its distinct evolutionary histories in the A- and B-genome components of cultivated peanut. Chromosom Res.

[9] Zhang L, Xu C, Yu W (2012). Cloning and characterization of chromosomal markers from a Cot-1 library of peanut (*Arachis hypogaea* L.). Cytogenet Genome Res.

[10] Du P, Li LN, Zhang ZX, Liu H, Qin L, Huang BY (2016). Chromosome painting of telomeric repeats reveals new evidence for genome evolution in peanut. J Integr Agr.

[11] Zhang LN, Yang XY, Tian L, Chen L, Yu WC (2016). Identification of peanut (*Arachis hypogaea*) chromosomes using a fluorescence in situ hybridization system reveals multiple hybridization events during tetraploid peanut formation. New Phytol.

[12] Cuadrado A, Golczyk H, Jouve N (2009). A novel, simple and rapid nondenaturing FISH (ND-FISH) technique for the detection of plant telomeres. Potential use and possible target structures detected. Chromosom Res.

[13] Beliveau BJ, Joyce EF, Apostolopoulos N, Yilmaz F, Fonseka CY, McCole RB (2012). Versatile design and synthesis platform for visualizing genomes with oligopaint FISH probes. Proc Nail Acad Sci USA.

[14] Mikš-Krajnik M, Babuchowski A (2014). 16S rRNA-targeted oligonucleotide probes for direct detection of Propionibacterium freudenreichii in presence of Lactococcus lactis with multicolour fluorescence in situ hybridization. Lett Appl Microbiol.

[15] Han YH, Zhang T, Thammapichai P, Weng YQ, Jiang JM (2015). Chromosome-specific painting in *Cucumis* species using bulked oligonucleotides. Genetics.

[16] Du P, Zhuang LF, Wang YZ, Yuan L, Wang Q, Wang DR (2017). Development of oligonucleotides and multiplex probes for quick and accurate identification of wheat and *Thinopyrum bessarabicum* chromosomes. Genome.

[17] Zhu MQ, Du P, Zhuang LF, Chu CG, Zhao H, Qi ZJ (2017). A simple and efficient non-denaturing FISH method for maize chromosome differentiation using single-strand oligonucleotide probes. Genome.

[18] Cuadrado A, Jouve N (2010). Chromosomal detection of simple sequence repeats (SSRs) using nondenaturing FISH (ND-FISH). Chromosoma.

[19] Wang YZ (2013). Development and characterization of small segment translocations of *Thinopyrum bessarabicum* and cytological mapping of interest genes.

[20] Tang ZX, Yang ZJ, Fu SL (2014). Oligonucleotides replacing the roles of repetitive sequences pAs1, pSc119.2, pTa-535, pTa71, CCS1, and pAWRC.1 for FISH analysis. J Appl Genet.

[21] Fu SL, Chen L, Wang YY, Li M, Yang Z (2015). Oligonucleotide probes for ND-FISH analysis to identify rye and wheat chromosomes. Sci Rep.

[22] Beliveau BJ, Joyce EF, Apostolopoulosa N, Yilmaza F, Fonseka CY, McCole RB (2013). Oligopaints: highly efficient, bioinformatically designed probes for fluorescence in situ hybridization. Epigenet. Chromatin.

[23] Danilova TV, Friebe B, Gill BS (2012). Single-copy gene fluorescence in situ hybridization and genome analysis: *Acc-2* loci mark evolutionary chromosomal rearrangements in wheat. Chromosoma.

[24] Li KP, Wu YX, Zhao H, Wang Y, Lu XM, Wang JM (2016). Cytogenetic relationships among *Citrullus* species in comparison with some genera of the tribe *Benincaseae* (*Cucurbitaceae*) as inferred from rDNA distribution patterns. BMC Evol Biol.

[25] Schimak MP, Kleiner M, Wetzel S, Liebeke M, Dubilier N, Fuchs BM (2016). MiL-FISH: multilabeled oligonucleotides for fluorescence in situ hybridization improve visualization of bacterial cells. Appl Environ Micro.

[26] Beliveau BJ, Boettiger AN, Avendaño MS, Jungmann R, McCole RB, Joyce EF (2015). Single-molecule super-resolution imaging of chromosomes and in situ haplotype visualization using oligopaint FISH probes. Nat Commun.

[27] Chen KH, Boettiger AN, Moffitt JR, Wang SY, Zhuang XW (2015). Spatially resolved, highly multiplexed RNA profiling in single cells. Science.

[28] Moffitta JR, Hao JJ, Wang GP, Chen KH, Babcock HP, Zhuang XW (2016). High-throughput single-cell gene-expression profiling with multiplexed error-robust fluorescence in situ hybridization. Proc Nail Acad Sci USA.

[29] Bertioli DJ, Cannon SB, Froenicke L, Huang GD, Farmer AD, Cannon EK (2016). The genome sequences of *Arachis duranensis* and *Arachis ipaensis*, the diploid ancestors of cultivated peanut. Nat Genet.

[30] Zhao CZ, Qiu JJ, Agarwal G, Wang JS, Ren XZ, Xia H (2017). Genome-wide discovery of microsatellite markers from diploid progenitor species, *Arachis duranensis* and *A. ipaënsis*, and their application in cultivated peanut (*A. hypogaea*). Front Plant Sci.

[31] Seijo G, Lavia GI, Fernández A, Krapovickas A, Ducasse DA, Bertioli DJ (2007). Genomic relationships between the cultivated peanut (*Arachis hypogaea*, Leguminosae) and its close relatives revealed by double GISH. Am J Bot.

[32] Koppolu R, Upadhyaya HD, Dwivedi SL, Hoisington DA, Varshney RK (2010). Genetic relationships among seven sections of genus *Arachis* studied by using SSR markers. BMC Plant Biol.

[33] Mallikarjuna N (2005). Production of hybrids between *Arachis hypogaea* and *A. chiquitana* (section *Procumbentes*). Peanut Sci.

[34] He L, Liu J, Torres GA, Zhang HQ, Jiang JM, Xie CH (2013). Interstitial telomeric repeats are enriched in the centromeres of chromosomes in *Solanum* species. Chromosom Res.

[35] Marcais G, Kingsford C (2011). Lock-free approach for efficient parallel counting of occurrences of *k-mers*. Bioinformatics.

[36] PeanutBase. https://peanutbase.org/gbrowse_aradu1.0. Accessed 22 Nov 2017.

[37] RepeatMasker. http://www.repeatmasker.org. Accessed 28 Nov 2017.

[38] Kent WJ (2002). BLAT - the BLAST-like alignment tool. Genome Res.

[39] Untergasser A, Cutcutache I, Koressaar T, Ye J, Faircloth BC, Remm M (2012). Primer3 - new capabilities and interfaces. Nucleic Acids Res.

[40] Murgha YE, Rouillard JM, Gulari E (2014). Methods for the preparation of large quantities of complex single-stranded oligonucleotide libraries. PLoS One.

[41] Wang CT, Huang Y, Yang XD, Jiang Y, Zhang JC, Chen DX (2002). Isolation of DNA from peanut: comparison between modified CTAB and high salt, low pH methods. J Peanut Sci.

[42] Chen CY, Barkley NL, Wang ML, Holbrook CC, Dang PM (2014). Registration of purified accessions for the U.S. Peanut mini-Core germplasm collection. J Pl Registr.

[43] Krapovickas A, Gregory W (1994). Taxonomia del genero *Arachis* (Leguminosae). Bonplandia.

