# Supplementary figures and images for: High-resolution chromosome painting with repetitive and single-copy oligonucleotides in Arachis species identifies structural rearrangements and genome differentiation
Source: BMC Plant Biol. 2018 Oct 17;18:240. doi: 10.1186/s12870-018-1468-1 (PMC6192370; doi:10.1186/s12870-018-1468-1)

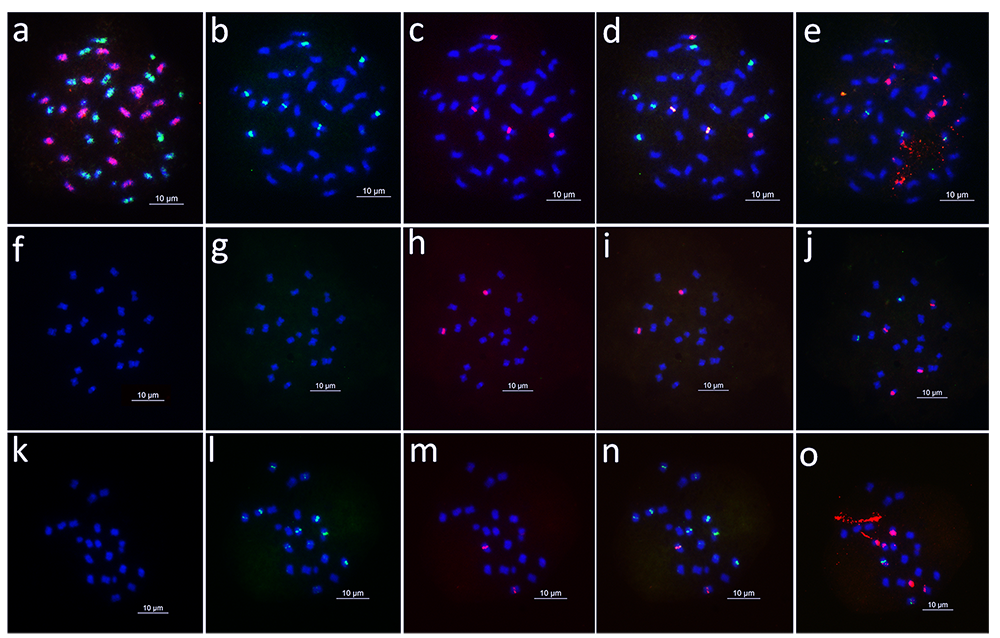

Supplement: Supplementary file 1 — Figure S1. FISH mapping of DP-2 (b, g, and l; green) and DP-1 (c, h, and m; red) by sequential FISH using 45S rDNA (e, g and j; red), 5S rDNA (e, g and j, green), and A. duranensis (a, green) and A. ipaënsis (a, red) total genomic DNA as probes in SLH (a–e), A. duranensis (f–j), and A. ipaënsis (k–o). (TIF 2370 kb) [file 12870_2018_1468_MOESM1_ESM.tif]

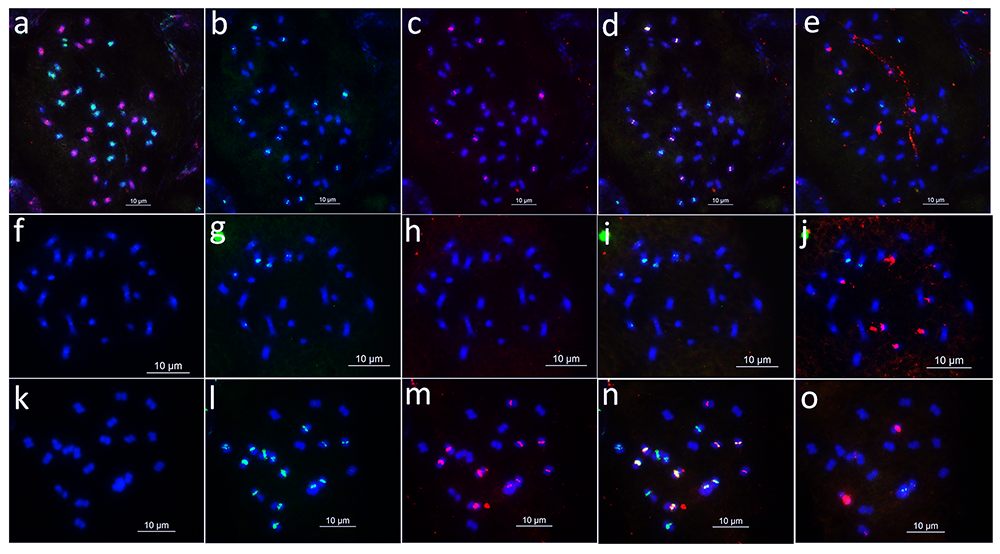

Supplement: Supplementary file 2 — Figure S2. FISH mapping of DP-3 (b, g, and l; green) and DP-4 (c, h, and m; red) by sequential FISH using 45S rDNA (e, g, and j; red), 5S rDNA (e, g, and j, green) and A. duranensis (a, green) and A. ipaënsis (a, red) total genomic DNA as probes in SLH (a–e), A. duranensis (f–j), and A. ipaënsis (k–o). (TIF 2188 kb) [file 12870_2018_1468_MOESM2_ESM.tif]

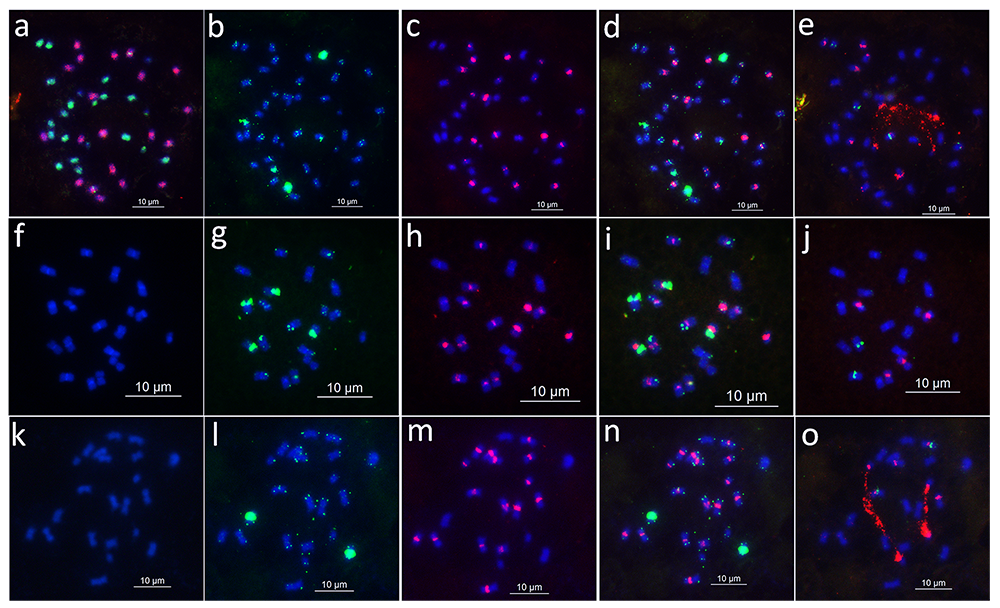

Supplement: Supplementary file 3 — Figure S3. FISH mapping of DP-5 (b, g, and l; green) and DP-6 (c, h, and m; red) by sequential FISH using 45S rDNA (e, g, and j; red), 5S rDNA (e, g, and j, green) and A. duranensis (a, green) and A. ipaënsis (a, red) total genomic DNA as probes in SLH (a–e), A. duranensis (f–j), and A. ipaënsis (k–o). (TIF 2587 kb) [file 12870_2018_1468_MOESM3_ESM.tif]

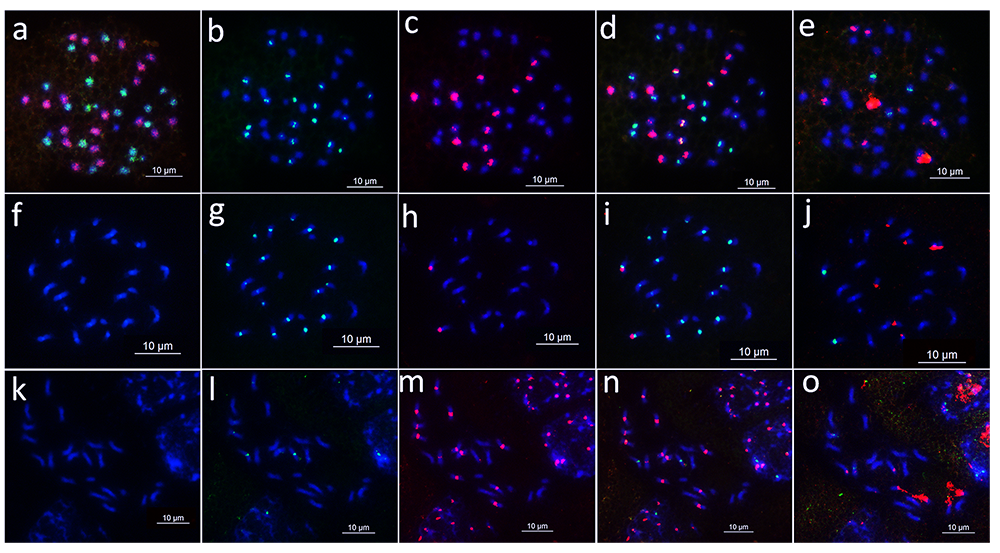

Supplement: Supplementary file 4 — Figure S4. FISH mapping of DP-8 (b, g, and l; green) and DP-7 (c, h, and m; red) by sequential FISH using 45S rDNA (e, g, and j; red), 5S rDNA (e, g, and j, green) and A. duranensis (a, green) and A. ipaënsis (a, red) total genomic DNA as probes in SLH (a–e), A. duranensis (f–j), and A. ipaënsis (k–o). (TIF 2283 kb) [file 12870_2018_1468_MOESM4_ESM.tif]

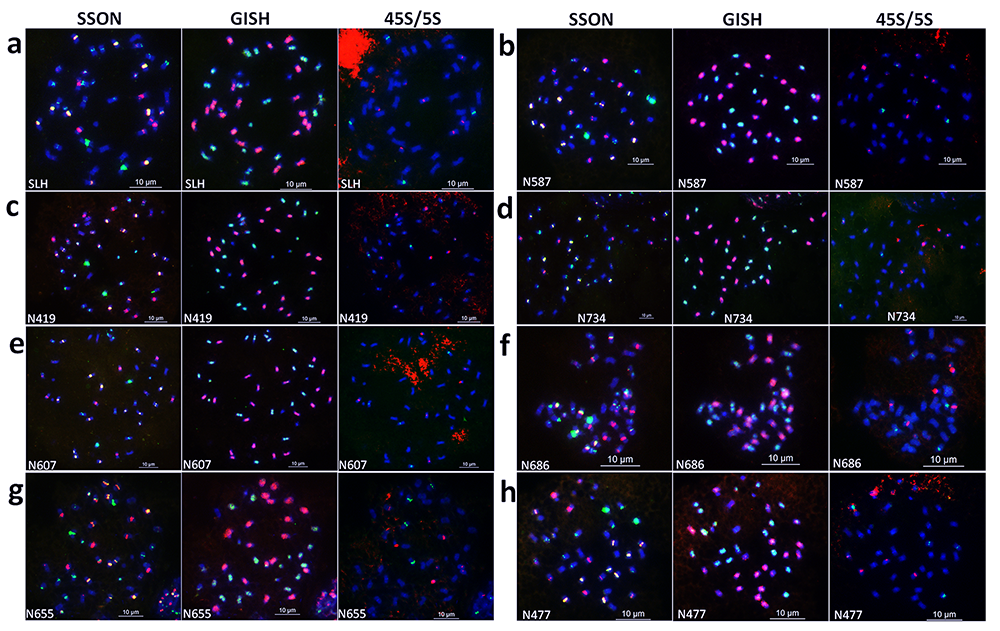

Supplement: Supplementary file 5 — Figure S5. Sequential FISH/GISH using Multiplex #1 (left column, SSON), A. duranensis genomic DNA, A. ipaënsis genomic DNA (middle column, GISH), 45S rDNA, and 5S rDNA (right column, 45S/5S) as probes in eight peanut varieties. (TIF 2593 kb) [file 12870_2018_1468_MOESM5_ESM.tif]

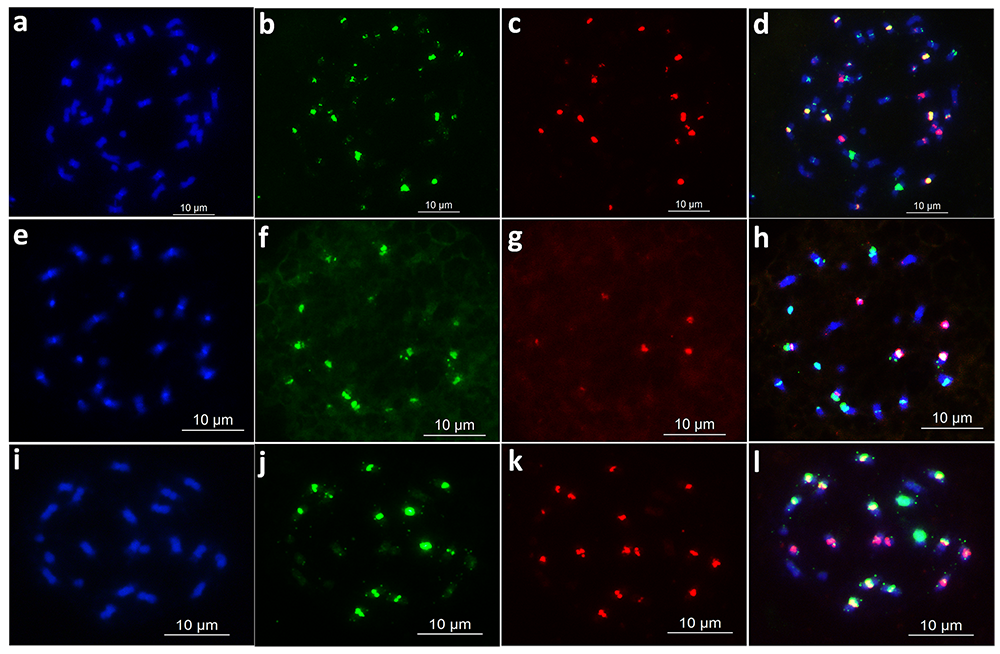

Supplement: Supplementary file 6 — Figure S6. Dual-color FISH using Multiplex #1 in SLH (a ~ d), A. duranensis (e ~ h), and A. ipaënsis (i ~ l). (TIF 1882 kb) [file 12870_2018_1468_MOESM6_ESM.tif]

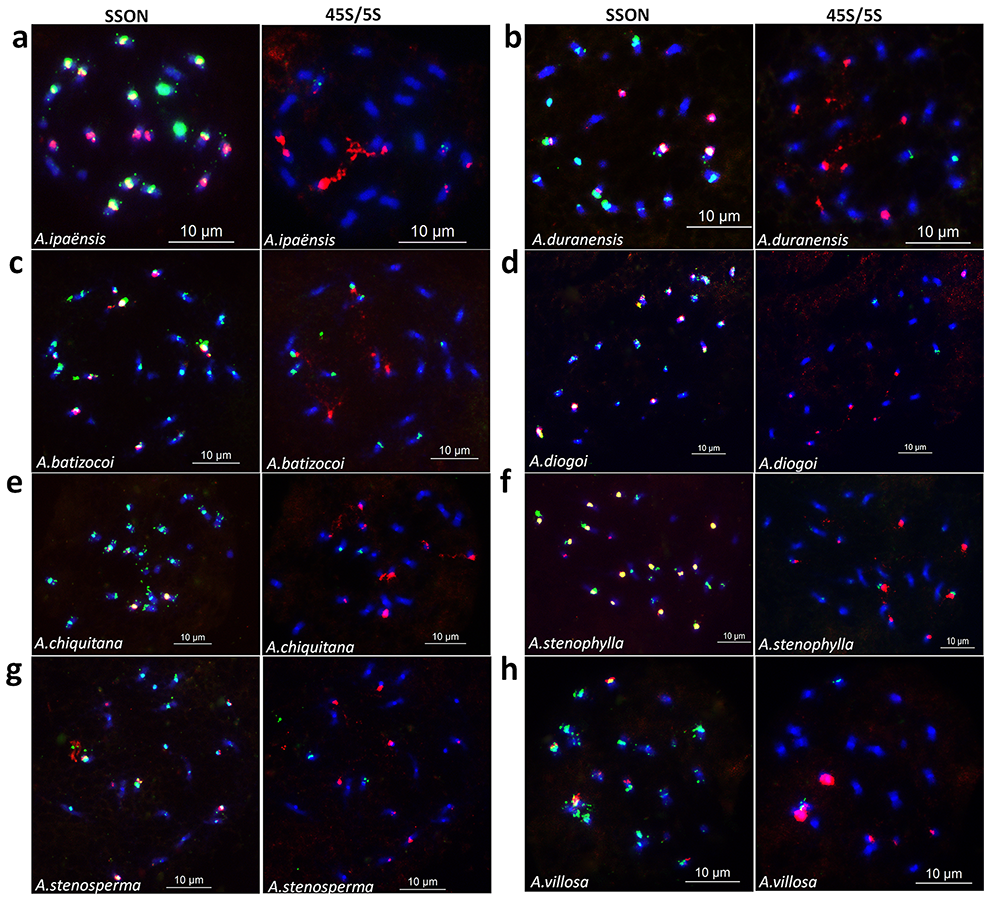

Supplement: Supplementary file 7 — Figure S7. Sequential FISH using multiplex #1 (left column, SSON), 45S rDNA, and 5S rDNA (right column, 45S/5S) as probes in eight Arachis species. (TIF 3606 kb) [file 12870_2018_1468_MOESM7_ESM.tif]

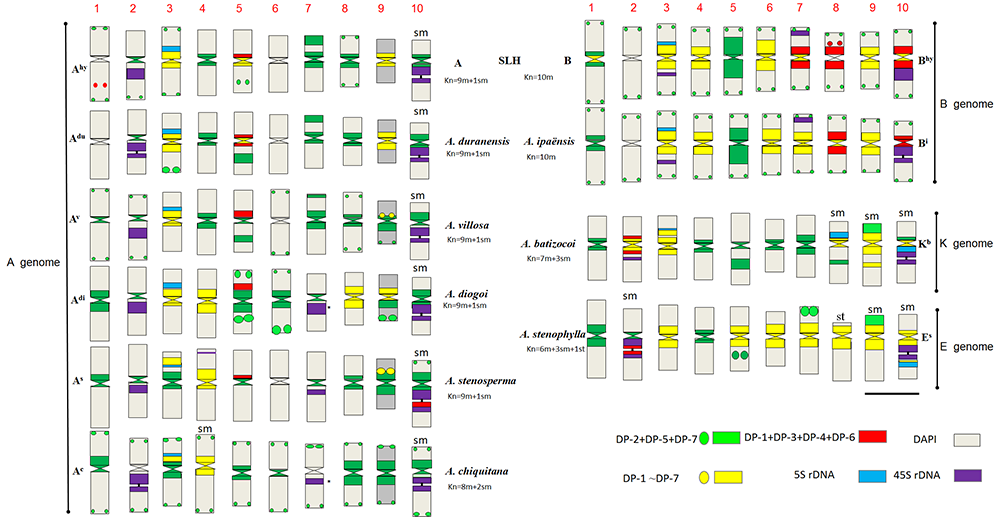

Supplement: Supplementary file 8 — Figure S8. Idiogram karyotypes of SLH and eight Arachis species. sm, submetacentric; st, subtelocentric; Bar, 3 μm. (TIF 666 kb) [file 12870_2018_1468_MOESM8_ESM.tif]

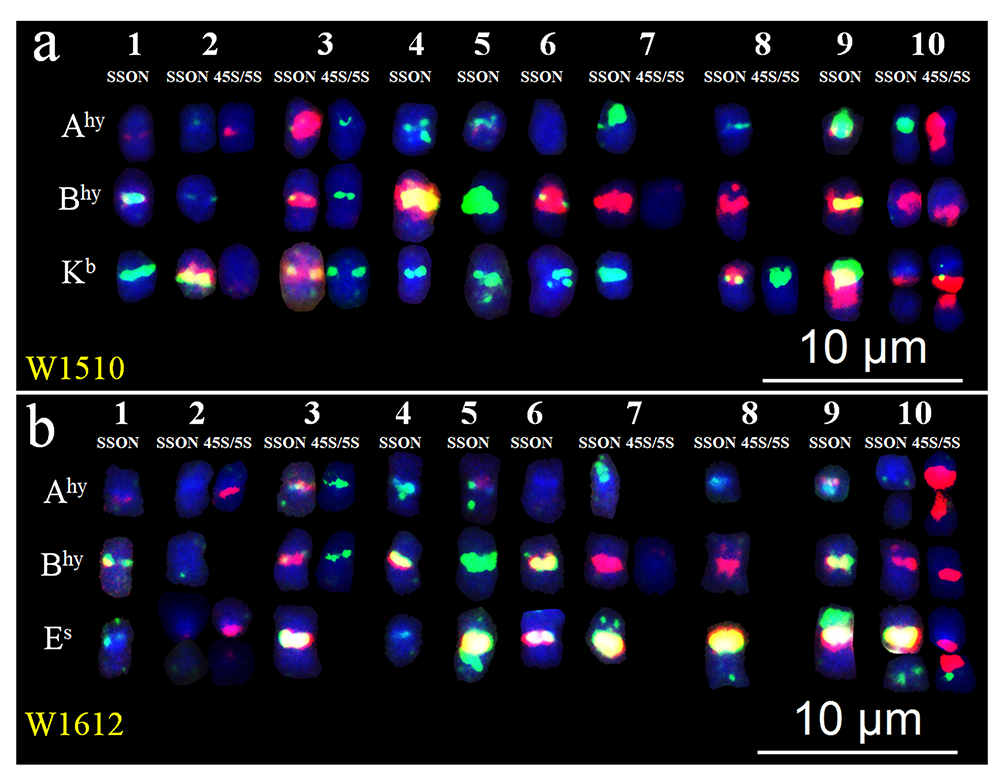

Supplement: Supplementary file 9 — Figure S9. Karyotypes of two hybrid F1-derived cultivated peanut and diploid wild species using repetitive Multiplex #1 (SSON), 45S and 5S rDNA (45S/5S) plasmid clones as probes. (TIF 1258 kb) [file 12870_2018_1468_MOESM9_ESM.tif]

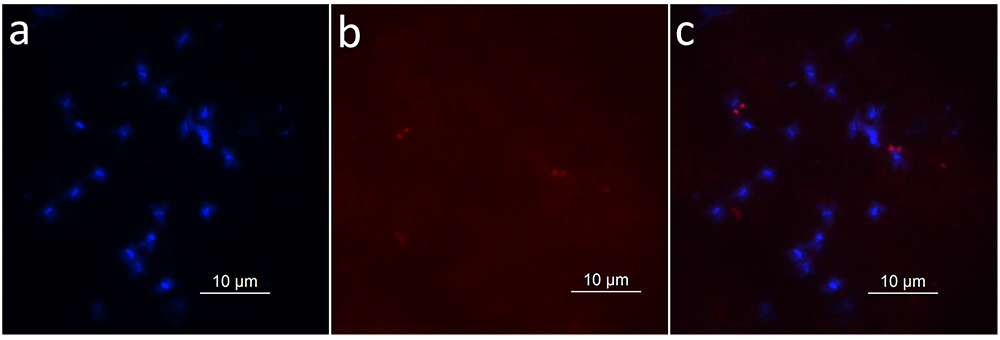

Supplement: Supplementary file 10 — Figure S10. FISH using oligonucleotide library 6A-1 in A. duranensis. (TIF 1066 kb) [file 12870_2018_1468_MOESM10_ESM.tif]

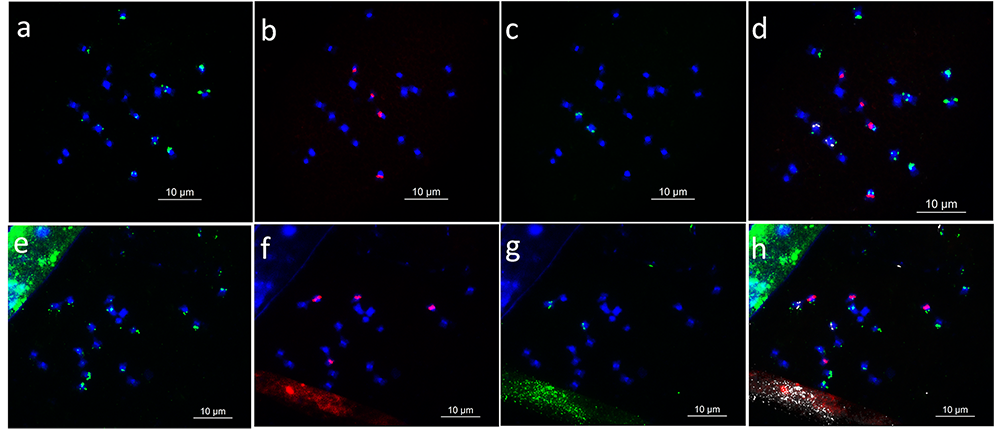

Supplement: Supplementary file 11 — Figure S11. Sequential FISH using DP-5 (a and e, green), 45S rDNA (b and f, green), and 5S rDNA (c and g, green) in A. duranensis, and its merged figures (d and h). (TIF 1141 kb) [file 12870_2018_1468_MOESM11_ESM.tif]
